# Solvent Effect in Imidazole-Based Poly(Ionic liquid) Membranes: Energy Storage and Sensing

**DOI:** 10.3390/polym13203466

**Published:** 2021-10-09

**Authors:** Arko Kesküla, Anna-Liisa Peikolainen, Paul A. Kilmartin, Rudolf Kiefer

**Affiliations:** 1 Intelligent Materials and Systems Lab, Institute of Technology, University of Tartu, Nooruse 1, 50411 Tartu, Estonia; arkokeskyla@gmail.com (A.K.); anna.liisa.peikolainen@ut.ee (A.-L.P.); 2 School of Chemical Sciences, The University of Auckland, Private Bag, Auckland 1142, New Zealand; p.kilmartin@auckland.ac.nz; 3 Conducting Polymers in Composites and Applications Research Group, Faculty of Applied Sciences, Ton Duc Thang University, Ho Chi Minh City 700000, Vietnam

**Keywords:** PIL films, solvents aq and PC, electroactivity, energy storage, sensor

## Abstract

Polymerized ionic liquids (PILs) are interesting new materials in sustainable technologies for energy storage and for gas sensor devices, and they provide high ion conductivity as solid polymer electrolytes in batteries. We introduce here the effect of polar protic (aqueous) and polar aprotic (propylene carbonate, PC) electrolytes, with the same concentration of lithium bis(trifluoromethane) sulfonimide (LiTFSI) on hydrophobic PIL films. Cyclic voltammetry, scanning ionic conductance microscopy and square wave voltammetry were performed, revealing that the PIL films had better electroactivity in the aqueous electrolyte and three times higher ion conductivity was obtained from electrochemical impedance spectroscopy measurements. Their energy storage capability was investigated with chronopotentiometric measurements, and it revealed 1.6 times higher specific capacitance in the aqueous electrolyte as well as novel sensor properties regarding the applied solvents. The PIL films were characterized with scanning electron microscopy, energy dispersive X-ray, FTIR and solid state nuclear magnetic resonance spectroscopy.

## 1. Introduction

Polymerized ionic liquids (PILs), as a new class of polymer electrolytes [1,2], have shown considerable potential for applications in batteries [3], antibacterial properties for biomedical devices [4], supercapacitors [5] and electrochemical devices [1,6,7]. PILs can be divided into polyanionic and polycationic type PILs [8], which differ in their localized charges [9]. In most cases the polycationic type is applied and to neutralize the charge, counter ions need to be present. The counter ions affect both the thermal and ion conductivities [5] and due to the nonpolar backbones, the charge species tend to cluster and form aggregates [8]. PILs with bis(trifluoromethane) sulfonimide (TFSI^–^) anions have been applied in surfaces switchable from hydrophobic to hydrophilic [10], and the effect of their high hydrophobicity has been addressed recently during the spontaneous assembly of nanoparticles in aqueous solution [11]. Previous research [8] has defined PILs as ion conductive polymers, with the finding that the ionic conductivity can be increased if the flexibility of the imidazolium group is enhanced. Often, PILs in solid electrolytes are combined with conducting polymers for dye-sensitized solar cells [12] or as electrodes for electrochromic devices [1]. Conducting polymers, on the other hand, are already reported in various works as being considered biofuel cells as well as sensors [13,14] as well as being considered for supercapacitor in combination with graphite [15]. Recent work showed that PIL monomers can be polymerized electrochemically together with pyrrole, forming conductive coatings with increased ionic conductivity [16] while stable linear actuation in various potential ranges was demonstrated [17]. Therefore, a combination with conducting polymers and PIL might be as well a future direction to enhance capacitance and sensor characteristics. Copolymerization of PIL monomers with other polymers has been carried out to increase ionic conductivity in membranes [18] or as an ion selective source in biosensors targeting DNA [19] or other proteins, also sensing pH and trapping heavy metals [10]. Another application is the combination with polystyrene for n-type transistors [6].

In this work, we have performed electrochemical measurements to study the electroactivity in imidazole-based PIL films with TFSI^-^ as counterions, using different solvents, namely aqueous solvent (aq) and propylene carbonate (PC), with the same concentration of LiTFSI salt. The goal of this research is to determine how polar protic and polar aprotic solvents change the electroactivity of PIL film as well as investigate the energy storage potential of such materials and their sensing properties.

Material characterization was performed applying solid state NMR (ssNMR), FTIR and SEM on the PIL films. Electrochemical impedance spectroscopy (EIS) measurements in the different solvents were made to determine the ion conductivity. For electrochemical characterization of PIL films, cyclic voltammetry in the potential range of 0.65 V−0.60 V (Ag/AgCl) in LiTFSI-PC and LiTFSI-aq electrolytes were performed. Square potential steps at frequencies from 0.0025 to 0.1 Hz were applied to determine their charging/discharging properties as well as compare the diffusion coefficients in the different solvents. Chronopotentiometric measurements were performed on the PIL films to determine the specific capacitance (energy storage) and their sensing ability towards the different solvents employed.

## 2. Material and Methods

### 2.1. Materials and Chemicals

1-Bromohexane (99%) and 1-vinylimidazole (>99%) were purchased from Alfa Assar (Alfa Aesar GmbH & Co KG, Haverhill, MA, USA). Propylene carbonate (99.7%), lithium bis(trifluoromethylsulfonyl)imide (LiTFSI, 99%) and α,α^’^-azoisobutyronitrile (AIBN, 98%) were obtained from Sigma-Aldrich (Taufkirchen, Germany) and used as received. All other solvents, diethylether, acetonitrile, acetone and methanol, were HPLC grade and purchased from Sigma-Aldrich (Taufkirchen, Germany). Milli-Q+ water (high-resistance ultra-purified water, Tallinn, Estonia) was used as supplied.

### 2.2. Synthesis and Polymerization of Polymerizable Ionic Liquid (PIL) Monomer

The PIL monomer synthesis is shown in the Supplementary Information, with the reaction given in Scheme S1 with the ^1^HNMR signals shown in Figure S1, as has been reported in previous research [20]. The PIL monomer was poured into a Teflon mold and polymerized at 60 °C using an initiator (AIBN), with a reaction time of 36 h. The obtained PIL film was 80 ± 2 µm thick and was washed several times with ethanol to remove residual unreacted monomer and dried in an oven at 40 °C (2 mbar) for 24 h.

### 2.3. Electrochemical Characterization of PIL Polymers

Electrochemical impedance (EIS) spectroscopy (PARSTAT 2273 potentiostat FRA, Princeton Applied Research, Berwyn, PA, USA) with the software Power Suite (Cummins Inc., Columbus, IN, USA) were applied to the PIL polymer films in 0.1 M LiTFSI in aqueous and propylene carbonate solvents. Pieces of PIL polymers of average thickness of 80 ± 2 μm were placed between gold electrodes with a width of 1 cm and length of 1.5 cm (area 0.00015 m^2^) and were subjected to alternating current with frequencies between 2 MHz and 10 mHz. The ionic conductivity ($\sigma_{i}$) was calculated using Equation (1), where *w* is the thickness of the polymer samples, *Z_re_* the real part of the polymer’s impedance and *A* the area of the surface between electrodes and the polymer.

(1) $$\sigma_{i}=\frac{w}{Z_{re}\cdot A}$$

The electrical impedance of a material in an alternating current circuit is influenced by many factors, such as the ionic conductivity, structure and capacitive behavior of the material. The impedance consists of a real part and an imaginary part, but Equation (1) only requires a real number value. To separate the real part, the measured resistance values have to be plotted onto a Nyquist plot.

Modified scanning ionic conductance microscopy (mSICM) of the PIL film surface in LiTFSI-PC and LiTFSI-aq electrolyte in potential range 0.65 to −0.2 V was applied in conjunction with cyclic voltammetry (50 mV s^−1^) measurements. Single-barrel, micro pipettes with tip diameters of 5–10 μm were fabricated using a Sutter Instruments P-2000 laser (Novato, CA, USA) puller from 100 mm long, 2 mm outside diameter borosilicate glass capillaries (Harvard Apparatus, product number 30-0117 (Holliston, MA, USA). The working electrode (WE) was directly connected to the polymer in a three-electrode cell setup, with a platinum wire as the counter electrode (CE) inside the micro pipette and a Ag/AgCl wire as the reference electrode connected to the samples, shown in images of the measurement setup in Figure S2.

Electrochemical measurements of the PIL films of 1.5 cm length and 0.1 cm width (free length between upper and lower clamp was in range of 8 mm) were performed in 0.1 M LiTFSI-PC and 0.1 M LiTFSI-aq in a three-electrode setup. PIL film, as a working electrode, was stretched up to 5 μm to a lower clamp with a gold contact on both sides of PIL film; a platinum sheet was a counter electrode and Ag/AgCl (3 M KCl) was a reference electrode). An upper force sensor (TRI202PAD, Panlab (Barcelona, Spain) of an in-house muscle analyzer [21] included a connected potentiostat (Biologic PG581, Seyssinet-Pariset, France). Before the measurements commenced, the PIL film in a stretched position was equilibrated in the electrolyte for 12 h. Different electrochemical techniques such as cyclic voltammetry and chronamperometry (frequency range 0.0025 to 0.1 Hz) and chronopotentiometry were applied. To calculate the diffusion coefficients of each chronoamperogramm at each applied frequency, Equations (2) and (3) were applied [22].

(2) $$\ln\left[1-\frac{Q}{Q_{t}}\right]=-b\cdot t$$

$Q_{t}\;$ is the total charge consumed by the time *t*, calculated from the integration of the current–time curve (from the chronoamperometric experiments), and Q is the charge consumed at each time point. The diffusion coefficient *D* is included in the constant b, as given by Equation (2), where *h* is the thickness of the PIL films (80 µm).

(3) $$D=\frac{b\cdot h^{2}}{2}$$

Plotting $\ln\left[1-\frac{Q}{Q_{t}}\right]$ versus *t* (Equation (2)) gives the slope *b* [23], and with the thickness of the PIL films, the diffusion coefficient *D* can be calculated. The weight of the PIL films in dry state was in the range of 556.2 ± 48 μg. Chronopotentiometric measurements at constant charge ±9 C g^−1^ at various current densities j (±0.045 A g^−1^, ±0.09 A g^−1^, ±0.18 A g^−1^, ±0.45 A g^−1^, ±0.9 A g^−1^ and ±1.8 A g^−1^) at frequencies 0.0025 to 0.1 Hz were applied. The specific capacitance *C*_s_ was calculated applying Equation (4) [24] and involved the current density *j* divided by the slope obtained from discharging potential to time curves (after IR drop) at each chronopotentiogram.

(4) $$C_{s}=\frac{j}{-slope}$$

### 2.4. Material Characterizations

Fourier transform infrared spectroscopy (FTIR) measurements were performed using a Bruker ALPHA spectrometer. PIL films were analyzed over the range of 3200 to 400 cm^−1^. The ^13^C MAS-NMR spectra were recorded on a Bruker (Billerica, MA, USA) AVANCE-II spectrometer in 14.1 T magnet (^13^C resonance at 150.47 MHz) using an in-house MAS_NMR double resonance probe with 4 mm rotors. The PIL monomer of CV6ImTFSI and PIL films were powdered, mixed with insulating fine powder (silica gel L600) and rotated at 12.5 kHz. A jelly sample 3 was measured at 9.1 kHz spinning speed. In all cases, a standard cross polarization (CP) pulse sequence was used with 0.5 ms CP pulse and subsequent proton decoupling. SEM (Helios NanoLab 600, FEI) surface images of the PIL polymer were taken. PIL membranes at charging (5 min charging time at 0.65 V) and discharging (5 min at −0.6 V) were performed in aqueous and propylene carbonate electrolytes, dried in an oven and the ion content was determined with EDX spectroscopy (Oxford Instruments with X-Max 50 mm^2^ detector (Abingdon, UK)).

## 3. Results and Discussion

PILs polymers are often named polymer electrolytes and they attract various applications in ion-conductive polymers in batteries, CO_2_ capture materials and bio-related devices [25].

### 3.1. Characterization of PIL Polymer Films

#### 3.1.1. Structure of PIL Monomer and Polymer by SEM, ssNMR and FTIR Spectroscopy

**Figure 1:**
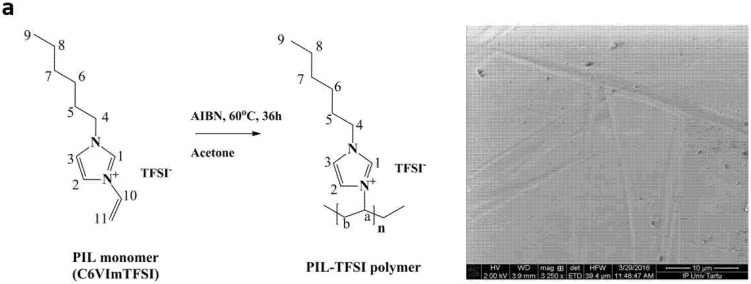

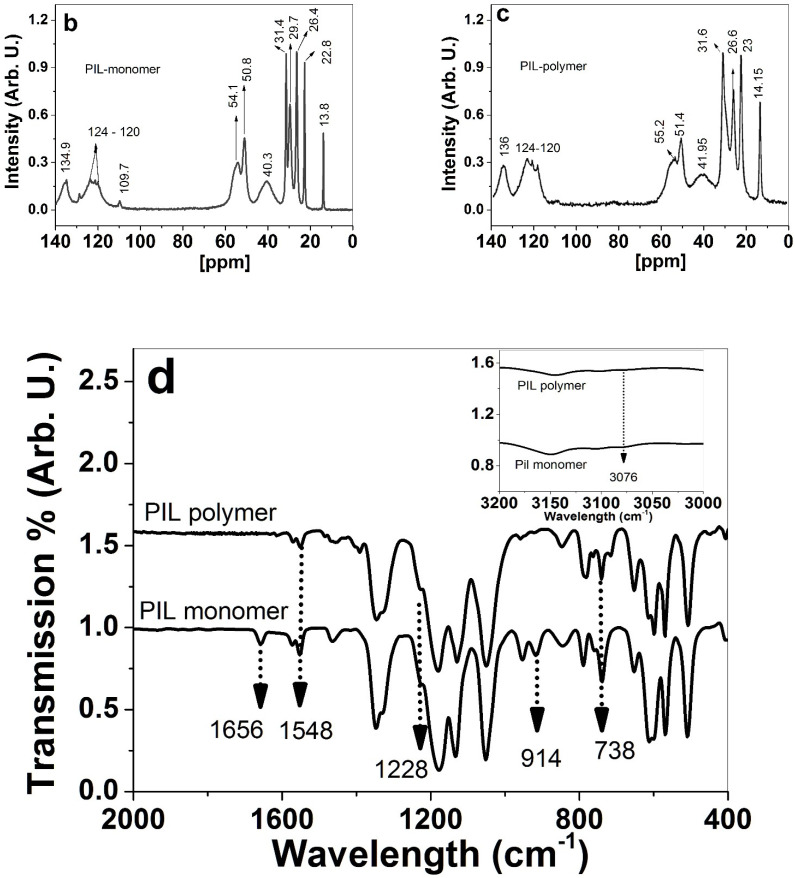
(**a**) C6VImTFSI monomer polymerization to PIL polymer with SEM surface image of PIL-TFSI polymer. The ^13^C MAS-NMR spectra (160 ppm to 0 ppm) of (**b**) PIL-monomer C6VImTFSI, (**c**) PIL polymer film and (**d**) FTIR spectroscopy from 2000 cm^−1^ to 400 cm^−1^, with inset 3200 to 3000 cm^−1^, for the PIL monomer and PIL polymer.

Figure 1a shows the polymerization reaction of the PIL monomer, along with an SEM image of the PIL polymer film. Solid state NMR (ssNMR) spectra of the PIL monomer and polymer are presented in Figure 1b,c, respectively, with FTIR spectra of both shown in Figure 1d. The PIL monomer CV6ImTFSI after chemical polymerization under heating with AIBN initiator (Figure 1a), revealed a yellowish glue-like form and a smooth surface as a film based on SEM images (Figure 1a inset) with good malleability in comparison to other initiators tried before. The long polymerization time with AIBN as initiator is advantageous in comparison with other initiators such as ammonium persulfate (APS). These faster initiators produce a rapid polymerization and with large amounts of initiator, the PIL films became brittle and glass-like and, in some cases, even foam-like structures which break easily.

The ssNMR spectroscopy of the PIL-monomer C6VImTFSI showed signals (Figure 1b) due to the vinyl group carbon C11 at 109.69 ppm (108 ppm in the literature [26]) which disappeared in the PIL polymer (Figure 1c), confirming that polymerization of the PIL monomer had taken place. From the imidazolium ring, the C1 atom (Figure 1a) can be found at 135 ppm (137 ppm in the literature [27]) and for the C2 and C3 of imidazolium, some peaks are located in the range of 120–124 ppm in both spectra’s (Figure 1b,c). Peaks at 40–55 ppm are related to the chain (hexyl group, C4-C8) and signals around 14–33 ppm include that of C9 at 14.2 ppm (methyl group), shown in Figure 1b,c [26]. The peaks of Ca and Cb groups of PIL polymer are shown in range of 40–60 ppm [26]. An additional peak at 29.68 is found in the PIL monomer but does not appear in PIL polymer, which we assume is overlapping with the broader peak at 31.6 ppm.

In the FTIR spectrum (Figure 1d), the 3076 cm^−1^ peak (=C-H stretching [10]) (inset in Figure 1d) shows that the vinyl group and the peak at 914 cm^−1^ represent the out of plane =C-H bending [10] of the PIL monomer which disappeared after polymerization, again confirming the polymerization of the PIL monomer. The peak at 1656 cm^−1^ that belongs to the C=C stretching band of the monomer [28] disappeared after polymerization while small peaks between 1550 and 1660 cm^−1^ shown in PIL polymer represented the C-N stretching vibration [10] of the imidazole ring as well as the peaks between 1250 and 1350 cm^−1^ that belong to the vibration of the C-N bond [29]. The signals from the counter anion TFSI^-^ appeared at 1228 cm^−1^ (shoulder) and 738 cm^−1^ (740 cm^−1^ in previous research [30]). From the results of FTIR and ssNMR, it can be concluded that the polymerization of the vinyl group of the PIL monomer has led to PIL polymer formation, and the TFSI^-^ as the counterion was also detected.

#### 3.1.2. EDX Spectroscopy and EIS Measurements of PIL Polymer Films

**Figure 2:**
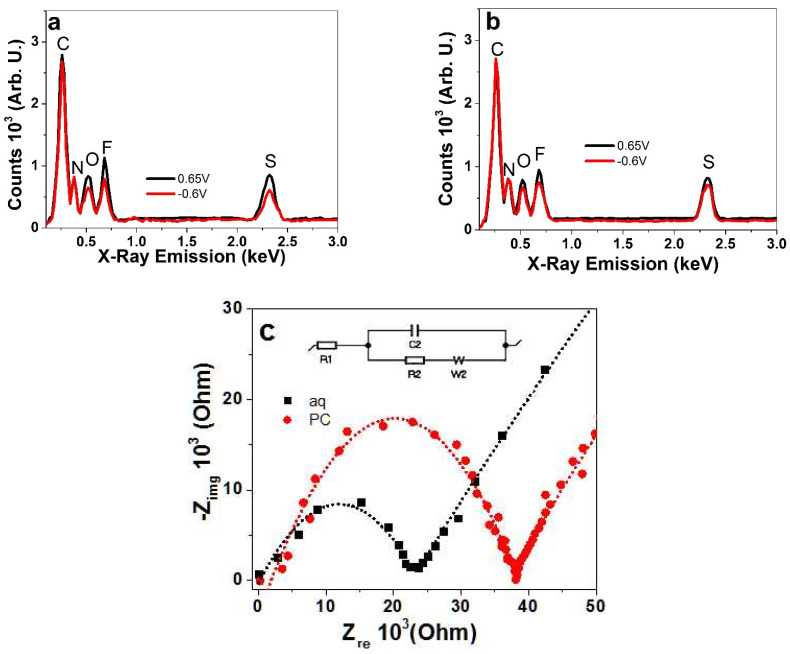
EDX spectroscopy of PIL polymer films at charged (0.65 V, black line) and discharged (−0.6 V, red line) state, applied in LiTFSI electrolyte with different solvents, (**a**) LiTFSI-aq and (**b**) LiTFSI-PC. (**c**) EIS Nyquist plots with fitting (dotted curve) (inset Randle’s equivalent circuit, R1: solution resistance, R2: charge transfer resistance, C2: double layer capacitance and W2: Warburg element) of PIL polymer in LiTFSI-aq (aq, ■) and LiTFSI-PC (PC, ●) electrolytes.

To investigate the ion content after charging/discharging cycles in LiTFSI-aq and LiTFSI-PC electrolytes for the PIL films, EDX spectroscopy was undertaken, and the results are presented in Figure 2a,b, respectively. The performance of PIL samples in different solvents with the same LiTFSI concentration were determined using EIS measurements, as presented in Nyquist plots in Figure 2c.

Figure 2a,b of the PIL polymers showed EDX peaks at 0.26 keV for carbon (C) and at 0.38 keV for nitrogen (N) from imidazolium ring and imide (Figure 1a). Signals from the TFSI^-^, counter ions that balance the localized positive charges, are found in the EDX peaks for oxygen (O) at 0.52 keV, fluoride (F) at 0.68 keV and sulfur (S) at 2.32 keV. Figure 2a shows the charging and discharging in aqueous LiTFSI electrolyte with nearly 1.4 times lower peak intensities at discharging, indicating that upon discharging the TFSI^-^ counter ions leave the PIL polymer film. If Li^+^ cations are incorporated during oxidation or reduction, this cannot be verified as Li^+^ cations could not be detected in the present EDX spectra. The EDX spectra of the PIL polymer in the LiTFSI-PC electrolyte, presented in Figure 2b, showed only a small decrease in peak intensities being around 1.2 times lower at discharging compared to charging. Thus, in different solvents, the ions that leave or remain inside the PIL films upon discharging are different. This depends on the physical properties of the solvent such as viscosity, ion mobility and conductivity, but also on the PIL film ion conductivity, which is analyzed through EIS measurements, as presented in Figure 2c.

From EIS measurements, the Z_re_ values were obtained with the Nyquist plots, from the 2nd intercept on the semi-circle; the values were obtained with a linear fit of Randle’s equivalent circuit shown in the inset of Figure 2c. The Z_re_ value of the PIL polymers in LiTFSI-PC was around 4052 ± 320 Ω, and that for the PIL polymers in LiTFSI-aq was at 1385 ± 98 Ω. From Equation (1), using area A (1.5 × 10^−4^ m^2^) and thickness w (8 × 10^−5^ m), the ion conductivity σ_I_ was calculated. The ion conductivity of PIL films in LiTFSI-PC at 0.13 ± 0.01 mS cm^−1^ was nearly three times smaller than that in the aq electrolyte, where an ion conductivity of 0.38 ± 0.026 mS cm^−1^ was obtained. In earlier research [8], it was discovered that polymerized ionic liquids behave as ion-conductive polymers but with a much lower conductivity compared to unpolymerized ionic liquids, in the range of 10^−4^ S cm^−1^ once the PILs are formed, similar to the present results. An increased ion conductivity can be obtained if flexible spacers [31] are included in the PIL monomer, which would allow the counter ions to move more freely. Research on pyrrolidinium nitrate protic ionic liquid in PC [32] was compared to those of aqueous pyrrolidinium hydrogen sulfate where a high ionicity was noted [33]; for PC, a negative effect appeared due to the ionicity of PC being lower than those of the pyrrolidinium nitrate samples. Therefore, the solvent plays a major role in the ion conductivity of PILs.

### 3.2. Electrochemical Characterization of PIL Films

Sensitive electrochemical characterization using cyclic voltammetry in micro-drops applying mSICM on the surface of the PIL films in aqueous and propylene carbonate-based electrolytes (with LiTFSI) was performed. Chronoamperometric studies at different frequencies, 0.0025 to 0.1 Hz, were also conducted.

#### 3.2.1. Cyclic Voltammetry of PIL Samples in mSICM on Surface and Films

**Figure 3:**
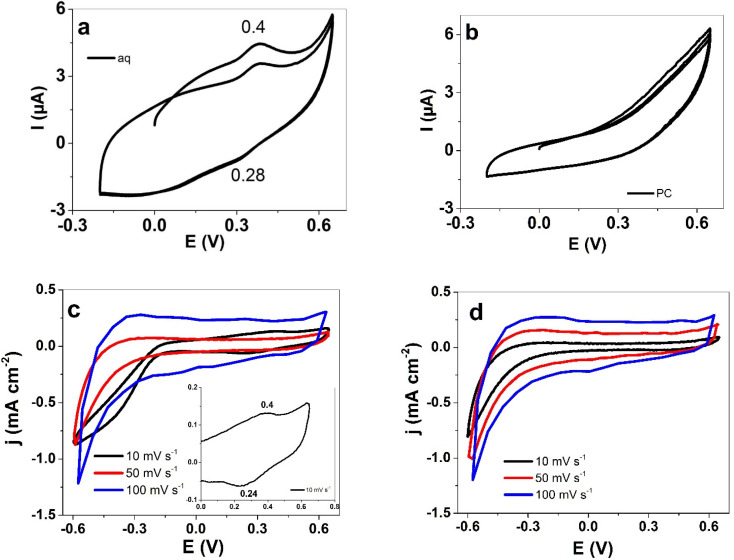
Modified SICM methodology during cyclic voltammetric measurements (scan rate 50 mV s^−1^) of surface of PIL samples at a potential range 0.65 V to −0.2 V using (**a**) LiTFSI-aq (aq) and (**b**) LiTFSI-PC (PC) as electrolytes. Cyclic voltammetry (3rd to 4th cycle) of PIL films at different scan rates 10 mV s^−1^ (black line), 50 mV s^−1^ (red line) and 100 mV s^−1^ (blue line) are depicted as current density j curves against potential E using (**c**) LiTFSI-aq (aq) (the inset depicts the curve at 10 mV s^−1^ scan rate at potential section from 0.65 V to 0.0 V) and (**d**) LiTFSI-PC (PC) as electrolytes.

Cyclic voltammetric measurements (scan rate 50 mV s^−1^) of PIL surfaces in LiTFSI-PC and LiTFSI-aq over the potential range from 0.65 to −0.2 V are shown in Figure 3a,b, respectively. The charge to potential curves from mSICM measurements are shown in Figure S3a. The associated charge density to potential curves from 0.65 to −0.6 V are presented in Figure S3b. The different potential ranges of mSICM were chosen due to the high sensitivity of the methodology, which appeared to cause irreversible reaction at lower potential range as well as at a lower scan rate: a longer time period led to evaporation of electrolyte solvent.

The cyclic voltammetric measurements in aq electrolyte (Figure 3a) revealed a charging peak at 0.4 V and a discharging wave at 0.32 V. The shape of the cyclic voltammogram in the PC based electrolyte showed no distinguishable charging nor discharging peaks. PIL films revealed for scan rate 10 mV s^−1^ in Figure 3c (inset), in aq electrolyte, a similar charging wave at 0.4 V and a small discharging wave at 0.24 V. Redox waves at this narrow potential range may indicate the polymerization of residue of PIL monomers in the PIL films during cycling. Figure S4 shows the potentiodynamic polymerization of PIL monomers (C6VImTFSI, Figure 1a) in LiTFSI-PC electrolyte where the oxidation peak at 1.1 V is related to the oxidation of the vinyl group of the PIL monomer, seen as well in recent research [34], with oxidation peak found in similar range of 0.85 to 1.0 V (the shifts relate to the different electrolyte and solvent applied). Earlier research [35] found out that solid electrolytes based on poly(vinylidene fluoride-hexafluoropropylene) (PVDF-HFP) and polyethylene glycol dimethyl ether (PEGDME) as well as PIL with TFSI moieties [36] have reversible redox properties, which were ascribed to impurities such as oxides on the electrode surfaces. We assume that the reversible redox reaction observed in Figure 3c is related to impurities. Higher scan rates in both solvents revealed similar cyclic voltammogram shapes with no further charging or discharging waves shown, and both solvents showed a discharging curve.

The mechanisms of charging/discharging in PIL films are not fully understood, and faradaic processes are questionable because there are no redox reactive compounds inside the PIL films. However, capacitive process has been described in recent research using PIL fiber-based material in pressure sensor textiles [37]. The large discharge curves for both solvents (Figure 3c,d) indicate increasing ion movement over electrostatic interactions [38], controlled by PIL polymer segmental motions influencing the ion transport [39] which is mainly influenced by the choice of counter ions [40].

In summary, the cyclic voltammetry of the PIL polymer film surface, as well as PIL films via mSICM (Figure 3a,b), revealed electrochemical activity [12] and ion transport [41].

#### 3.2.2. Square Potential Steps Measurements

**Figure 4:**
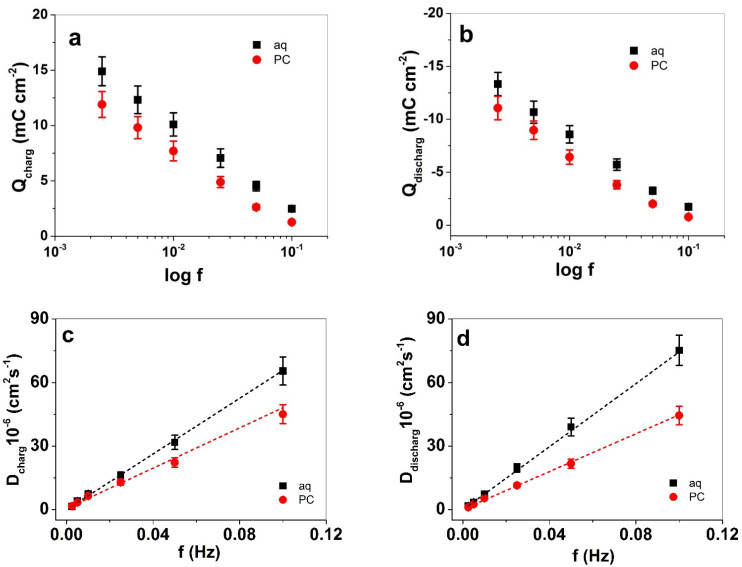
Square potential steps analysis of PIL films in LiTFSI-aq (aq, ■) and LiTFSI-PC (PC, ●) at applied potential range 0.65 V to −0.6 V, and applied frequencies from 0.0025 Hz to 0.1 Hz. (**a**) Charge densities at charging Q_charg_, (**b**) charge densities at discharging Q_discharg_ against logarithmic scale of frequencies f, (**c**) diffusion coefficients at charging, D_charg_, and (**d**) at discharging D_discharg_ against applied frequencies f. The dashed black and red lines refer to the linear fit shown here only for orientation.

The charging/discharging properties of PIL films in LiTFSI-aq and LiTFSI-PC electrolytes were characterized using square potential steps analysis at different frequencies from 0.0025 to 0.1 Hz, with results shown in Figure 4a,b, respectively. The diffusion coefficients at charging and discharging, calculated according to Equations (2) and (3), are presented in Figure 4c,d. The current density to time curves for a frequency of 0.005 Hz are shown in Figure S4.

The charge density of PIL films were obtained over integration of current density curves at each frequency (for example, the frequency of 0.0025 Hz in Figure S5 reveals the capacitive nature of PIL), shown in Figure 4a,b. At each applied frequency, the charge density at charging and discharging was 1.2 times higher in aq LiTFSI solution than in the case of LiTFSI-PC. In previous research [42], it has been reported that solvent plays an important factor in the structure of PIL materials: pores form in aqueous solution due to shrinking of the copolymer at the interface between the aqueous and copolymer domains. As a consequence, the PIL film collapses, forming an unswollen film, whereas the mobility of the anion TFSI^−^ is enhanced due to limited solvation shell [43], allowing them to move more freely in and out of the PIL film. In contrast to PC, where each TFSI^−^ anion is accompanied by both Li^+^ cations and PC molecules, neutral complexes or clusters are formed if a higher concentration of electrolyte is applied [44].

It is well known that imidazolium groups with TFSI^−^ anions have hydrophobic properties [45]. We assume that during the charging/discharging process, it is the non-swollen nature of PIL films in aqueous LiTFSI electrolyte solution which allows more ion exchange as well as higher ion diffusion (Figure 4c), with nearly 1.4 times higher diffusion coefficients at charging (D_charg_) and 1.7 times higher diffusion coefficients at discharging (D_discharg,_ Figure 4d) in comparison to PIL films in LiTFSI-PC at 0.1 Hz frequency. In propylene carbonate, the PIL films are swollen forming a separation between the imidazolium backbone of polycation and the TFSI^-^ anions [42], inducing a charge separation which decreases the ion diffusion.

### 3.3. Specific Capacitance of PIL Films Applied at Electrolytes with Different Solvents

**Figure 5:**
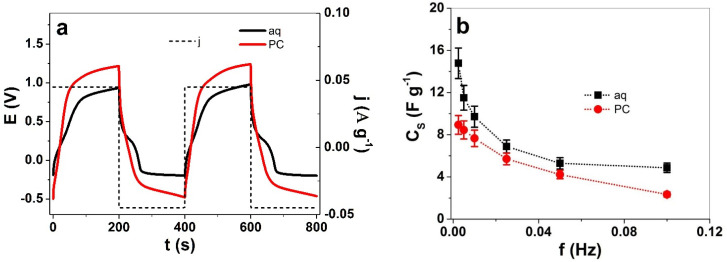
Chronopotentiograms of PIL films in LiTFSI-aq (aq, black curve) and in LiTFSI-PC (PC, red curve) at an applied frequency 0.0025 Hz. (**a**) Potential E to time curves (3rd and 4th cycle) with current densities j (dashed black line) and (**b**) the specific capacitance C_s_ for PIL films in LiTFSI-aq (aq, ∙∙■∙∙) and LiTFSI-PC (PC, ∙∙●∙∙) against applied frequencies f (0.0025 Hz to 0.1 Hz).

**Figure 6:**
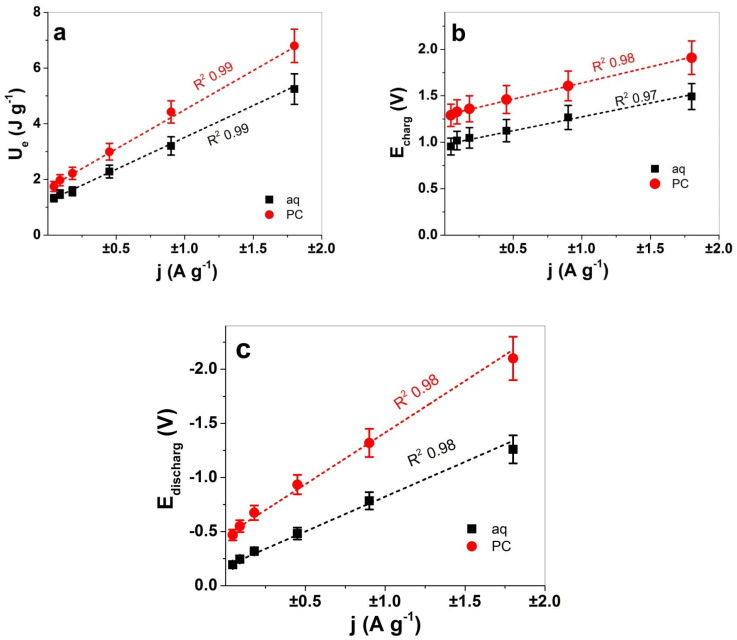
Chronopotentiometric measurement of PIL films in LiTFSI-aq (aq, ■) and LiTFSI-PC (PC, ●) electrolytes. (**a**) The consumed electrical energy U_e_, (**b**) the potential E_charg_ at charging and (**c**) the potential E_discharg_ at discharging against the current density j. The dashed black and red lines represent the linear fit including the correlation coefficient R^2^ 0.97 to 0.99.

The capacitance of PIL polymers is usually ascribed to the ion-conductive properties and the polarizable ions of the polymers [37]. The PIL film weight after measurements was determined in the dry state (556 ± 48 μg). The changes in weight if exposed to aqueous electrolyte showed a 5% increase in weight to 584 ± 51 μg and in the case of organic electrolyte, a nearly 40% increase (774 ± 67 μg) was found. The changes in weight are included in the following calculations. Chronopotentiometric measurements were performed to investigate how the solvent influenced the specific capacitance of the PIL films. Figure 5a presents the potential to time curves at an applied frequency of 0.0025 Hz, and the current density j of ± 0.045 A g^−1^ (constant charge of ± 9 C g^−1^), which was related to the weight of the PIL films in dry state. From Equation (4), the specific capacitance C_s_ was calculated, and the results are shown in Figure 6b.

The potential to time curves of PIL films, shown in Figure 5a, revealed nearly 1.4 times higher charging potential in the PC based electrolyte while the discharging in the LiTFSI-aq at the lowest point was found at −0.19 V and that of LiTFSI-PC at −0.47 V (nearly 2.5 times higher). The main reason for the differences between aq and PC solvents relies on the reduced ionic conductivity in the case of PC (three times lower, Figure 2c), which affects the capacitance of the PIL films. Taking the slope (after IR drop) from discharging potential to time curves at each frequency, the specific capacitance C_s_ was calculated (Equation (4)), as presented in Figure 5b. The highest specific capacitance of PIL films in this study found for LiTFSI-aq at 0.0025 Hz, was in the range of 14.9 ± 1.3 F g^−1^, being nearly 1.6 times higher than 8.9 ± 0.8 F g^−1^ determined in LiTFSI-PC (Figure 5b). There are only a few reports using PILs for applications in energy storage, such as PIL polymers cross-linked with polyethylene oxide (PEO) in the solid state. This showed a specific capacitance of 12 F g^−1^ [46] while the protic PIL polymers from 1-butylpyrrolidinium bis(trifluoromethanesulfonyl)imide, with few percentages of water, up to 3.8% [47], led to a specific capacitance in the range of 18 to 20 F g^−1^, which showed that the addition of water reduced the electrochemical stability window. In most cases, PILs applied with the addition of deposited polypyrrole in the solid state showed a pseudo capacitance in the range of 5 F g^−1^ [48], while direct polymerization of pyrrole with PIL monomers reached 74 F g^−1^ [20]. Due to the differences in capacitance and electrochemical stability in the case of different electrolyte solvents, the sensor properties of the PIL films was investigated.

### 3.4. Electrochemical Sensor Characteristics of PIL Films

Earlier research [49,50], mainly based on conducting polymer-based actuators, has shown that they can simultaneously have a sensor function in chronoamperometric studies, recording the change in charge, potential and reaction energy based on the Le Chatelier principle [51]. Given that PILs are ion-conductive polymers having ion mobility and capacitance, the sensor properties were investigated in both LiTFSI-aq and LiTFSI-PC. To obtain the electrical energy U_e_ consumed, at each chronopotentiogram and applied current density, the potential to time curves shown in Figure 5a were integrated at discharging and the obtained values were multiplied with current density j leading to electrical energy U_e_ (Equation (5)).

(5) $$U_{e}=j\int E\left(t\right)dt$$

Figure 6a compares the evolution of electrical energy of PIL film in the two solvents aq and PC. Another variable regarding the potential time curves (Figure 5a), at each applied frequency and current density, is the maximum potential at charging E_charg_ and discharging E_discharg_, which can be displaced against the applied current density j, as shown in Figure 6b,c, respectively.

Figure 6a showed differences in consumed energy U_e_ obtained from Equation (5) and includes linear fits for both solvents, which shows a good correlation coefficient R^2^ of 0.99. The PIL film in LiTFSI-PC had 1.3 times higher electrical energy in comparison to the LiTFSI-aq. From the linear fit in Figure 6a, the sensor properties of PIL films can be calibrated to obtain two different expressions, one for LiTFSI with an aq solvent and one for LiTFSI with PC as a solvent. Other parameters such as the potential evolution and its dependence on the applied current density are shown for PIL films, where a clear separation of potential values between aq and PC solvents was shown. From the linear fit (correlation coefficients R^2^ from 0.97 to 0.98) of the potential at charging (Figure 6b) and discharging (Figure 6c), linear equations (obtained from slopes) of PIL films in aqueous LiTFSI-aq and organic LiTFSI-PC electrolyte solutions can be formulated. The sensor equations obtained from Figure 6a–c of the consumed electrical energy U_e_, the potential at charging E_charg_ and discharging E_discharg_ are compared in Table 1 for LiTFSI-aq and LiTFSI-PC.

The linear sensor equations (Table 1), such as electrical energy U_e_ and potential at charging E_charg_, did not differ greatly between the two solvents. So far, the best parameter for sensing different solvents of the electrolyte was found to be the potentials at discharging, which showed a clear separation (nearly 1.5 times different) between aq and PC solvent-based electrolytes. The linear equations in Table 1 revealed that in an aqueous solvent-based electrolyte, the PIL films have reduced electrical energy and potentials at discharging in comparison to the PC solvent-based electrolyte. Therefore, PIL films are indeed applicable for sensor elements detecting different solvents such as the polar protic (aq) and polar aprotic (PC) used in this study. PILs certainly have potential as humidity sensors [25], making such materials interesting for healthcare applications. The storage capability of PIL membranes towards carbon dioxide [52] were investigated as sensor materials leading to CO_2_ gas detection by applying PILs with CNT materials [53]. More research is needed for alternative applied electrolytes and conditions, such as different electrolytes, solvents, temperatures and pressures which should be applied to verify the sensor sensibility and stability.

## 4. Conclusions

PILs, as a new class of polymer electrolytes, have recently shown great potential in various applications. The addition of electrolytes with good ion conductivities were investigated in this work. PIL films underwent electrochemical characterization in aqueous and propylene carbonate-based solutions of LiTFSI salt. The PIL films in the aqueous electrolyte solution showed much better electrochemical properties with ion conductivity (EIS measurements) 3 times higher, charge at charging/discharging 1.2 times higher and the diffusion coefficient at discharge 1.7 times higher than in the PC based electrolyte. The energy storage capability obtained over the specific capacitance was 1.6 times larger in the aqueous electrolyte and the novel sensor characteristics revealed that the different electrolyte and solvents can be determined based on electrical parameters obtained for PIL film. The results from this study will trigger new applications for PILs as electrochemical sensors and energy storage materials, applicable in healthcare and biomaterial devices.

## Figures and Tables

**Table 1:** Linear equation comparing the slopes of electrical energy U_e_, potential at charging E_charg_ and discharging E_discharg_ of PIL films in aq (aqueous) and PC (propylene carbonate) based electrolytes, with the same concentration of LiTFSI salt.

Solvents with LiTFSI	Linear Equation of Electrical Energy U_e_ [J g^−1^]	Linear Equation of Potential at Charging E_charg_ [V]	Linear Equation of Potential at Discharging E_discharg_ [V]
aq	$2.29\;\pm 0.07\;j\left(Ag^{-1}\right)$	$0.30\;\pm 0.02\;j\left(Ag^{-1}\right)$	$-0.64\;\pm 0.04\;j\left(Ag^{-1}\right)$
PC	$2.82\;\pm 0.12\;j\left(Ag^{-1}\right)$	$0.35\;\pm 0.01\;j\left(Ag^{-1}\right)$	$-0.96\;\pm 0.05\;j\left(Ag^{-1}\right)$

## Data Availability

The data presented in this study are available on request from the corresponding author.

## Supplementary Materials

The Supplementary Materials are available online at https://www.mdpi.com/article/10.3390/polym13203466/s1.
