# Supplementary material for: Solvent Effect in Imidazole-Based Poly(Ionic liquid) Membranes: Energy Storage and Sensing
Source: Polymers (Basel). 2021 Oct 9;13(20):3466. doi: 10.3390/polym13203466 (PMC8537087; doi:10.3390/polym13203466)
Supplement: Supplementary file 1 [file polymers-13-03466-s001.zip › polymers-1406368-supplementary.pdf]

polymers

MDPI

Supplementary

Solvent effect in imidazole-based poly(ionic liquid) membranes: energy storage and sensing

Arko Kesküla[1], Anna-Liisa Peikolainen[1], Paul A. Kilmartin[2] and Rudolf Kiefer[3,*]

[1]Intelligent Materials and Systems Lab, Institute of Technology, University of Tartu, Nooruse 1, 50411 Tartu, Estonia
[2]School of Chemical Sciences, The University of Auckland, Private Bag, Auckland 1142, New Zealand
[3]Conducting polymers in composites and applications Research Group, Faculty of Applied Sciences, Ton Duc Thang University, Ho Chi Minh City, Vietnam
* Correspondence: author. Tel: +886 905605515. E-mail: rudolf.kiefer@tdtu.edu.vn (Rudolf Kiefer)

Synthesis of PIL monomer

1-vinyl-3-hexylimidazolium bromide (C6VImBr) was prepared from the N-alkylation of 1-vinylimidazole (1 eq.) with 1-bromohexane (1.1 eq.). Under dry nitrogen atmosphere and vigorous stirring 17.6 g of freshly distilled 1-bromohexane (106 mmol) was added drop-wise to 9.23 g of 1-vinylimidazole (98 mmol). The reaction mixture was kept at 60 °C under reflux for 24 hours. The mixture was cooled down to room temperature, where two liquid phases separated. The upper phase was removed by decantation, and the remaining brownish ionic liquid 1-vinyl-3-hexylimidazolium bromide (C6VImBr, Scheme S1) was washed three times with diethyl ether (3 × 30 ml) and then dried in a rotavapor. The product was further purified from an acetonitrile/diethyl ether mixture and finally dried under vacuum giving a 95.4% yield. The C6VimBr product was characterized by ^{1}H-NMR (400 MHz, DMSO, ppm) shown in Figure S1.

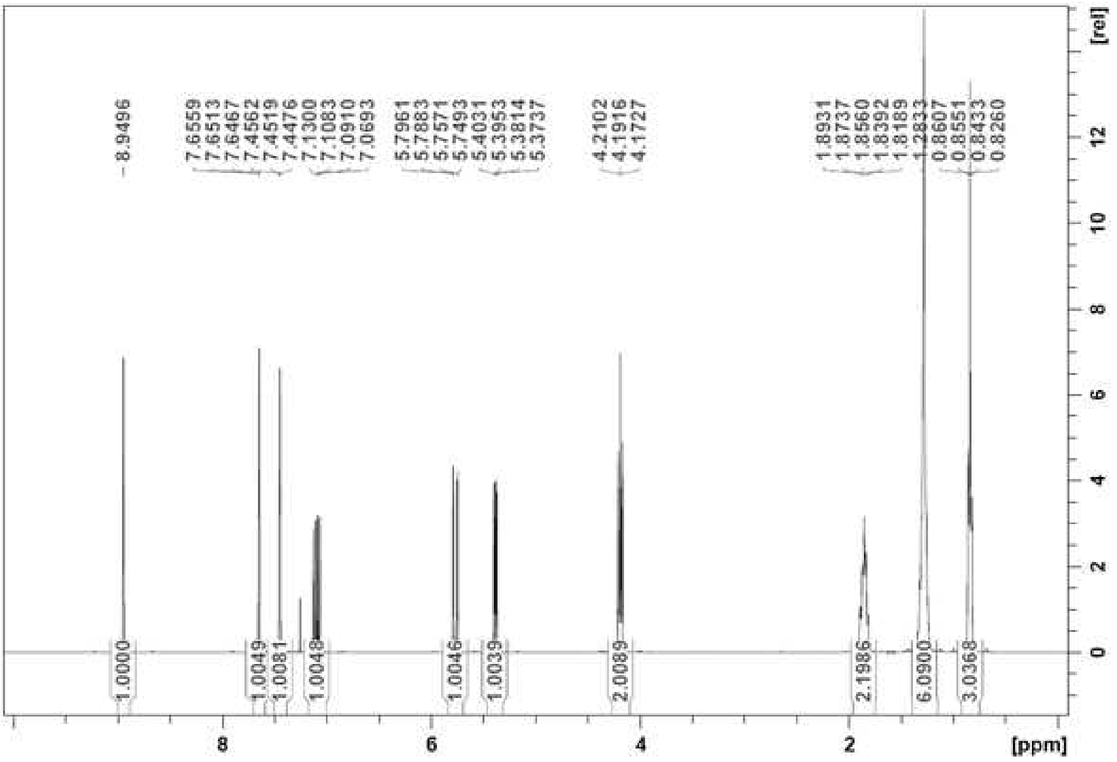

Figure S1. ^{1}H-NMR (400 MHz, DMSO, ppm] of 1-Vinyl-3-hexylimidazolium bromide (C6VImBr).

Signals (Figure S1) appearing for ^{1}H-NMR at 8.95 (s, 1H), 7.65 (s, 1H), 7.45 (s, 1H), 7.13 (s, 1H), 7.1-7.07 (m, 1H), 5.79-5.75 (m, 1H), 4.21-4.17 (t, 2H), 1.89-1.81 (m, 2H), 1.28 (s, 6H) and 0.86-0.82 (m, 3H). The final polymerizable ionic liquid 1-vinyl-3-hexylimidazolium TFSI (C6VImTFSI) was prepared by the ion metathesis of (C6VImBr) using LiTFSI. A solution of bis(trifluoromethylsulfonyl)imide lithium salt (3.58 g, 12 mmol) in 20 ml of deionized water was added dropwise to 3.16 g of 1-vinyl-3-hexylimidazolium bromide (12 mmol) dissolved in 60 ml of methanol.

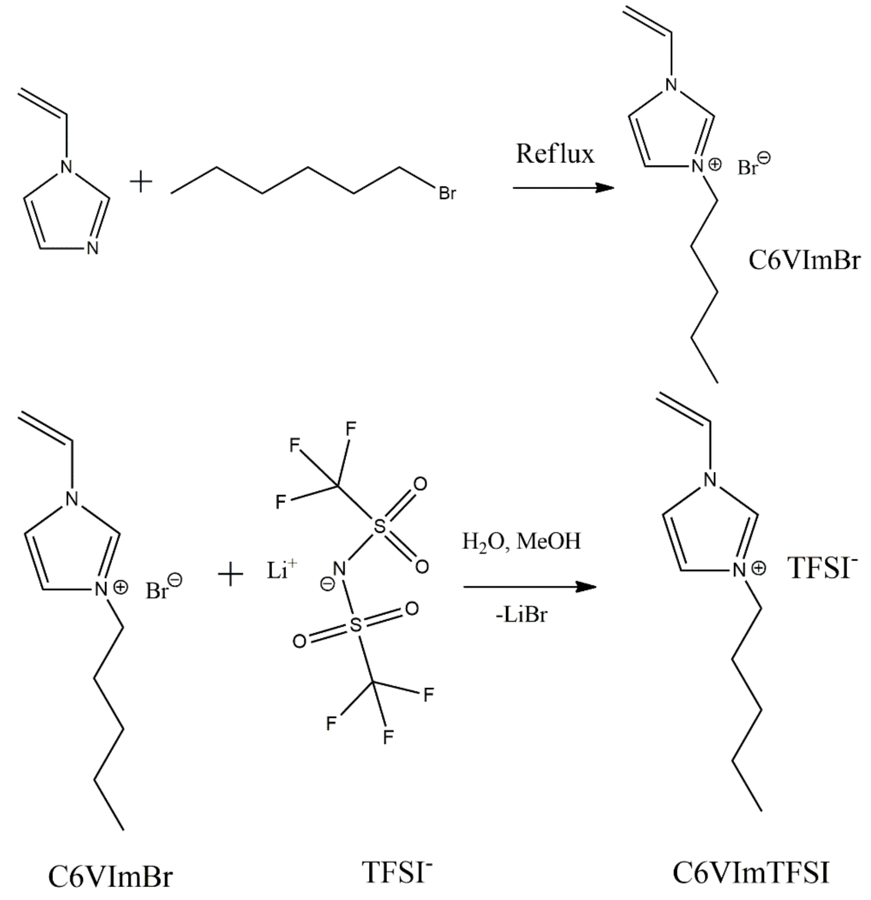

Scheme S1: Synthesis of C6VImTFSI (PIL monomer)

The reaction mixture was stirred at room temperature overnight, and then concentrated under reduced pressure to remove methanol. After decantation, the separation of a brown viscous liquid occurred, and it was washed three times with water. The ionic liquid was then dried in a rotavapor at 50 °C.

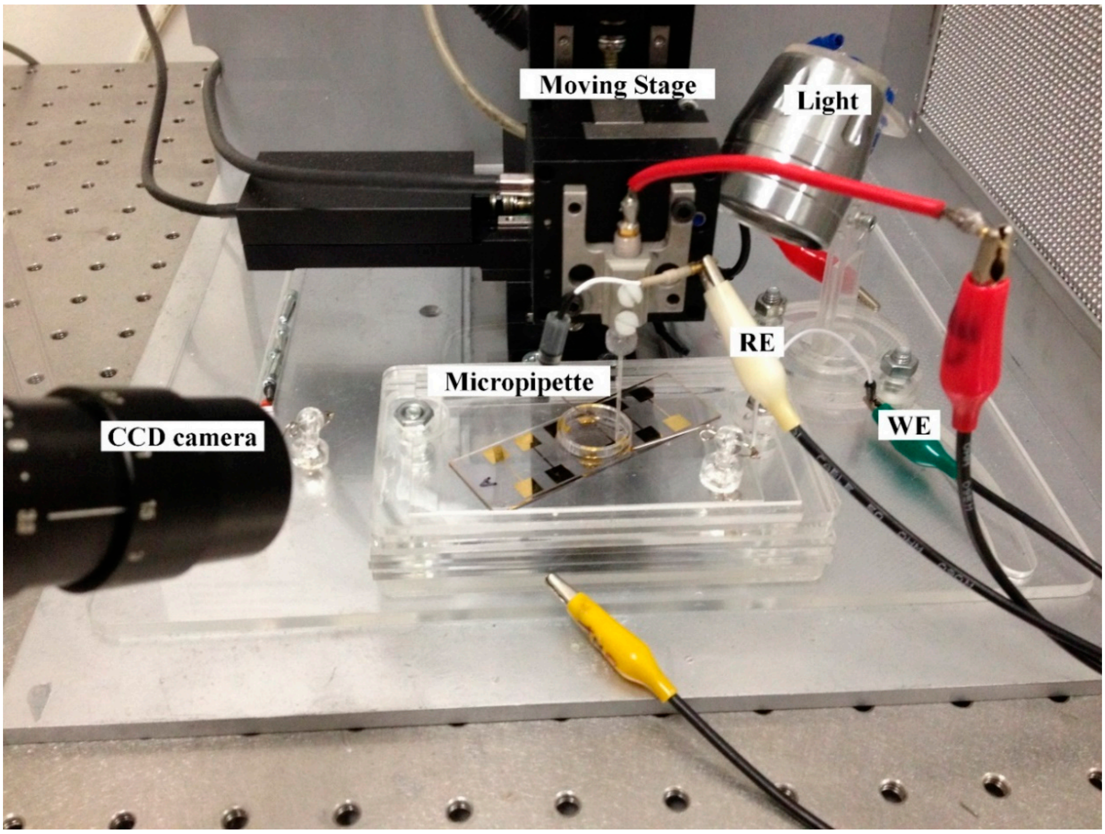

Figure S2. Modified SICM set up included a micropipette positioned over a CCD camera in the right position. Table S3. Coulovoltammetric curves of PIL films in LiTFSI-aq (aq, black curve) and LiTFSI-PC (PC, red curve) showing in a: the charge against potential of PIL films measured by mSICM on the surface; and b: the charge density against potential of bulk PIL films.

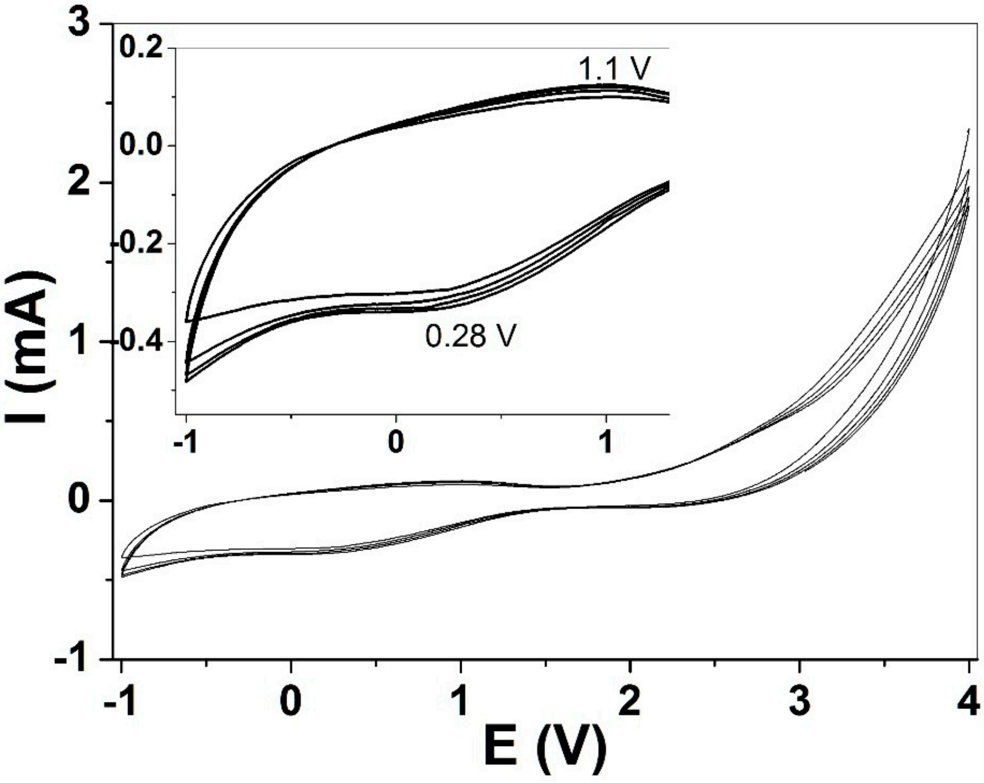

Figure S3. Potentiodynamic polymerization (scan rate 50 mV s^{-1}) of PIL monomer in LiTFSI in propylene carbonate at applied potential range of -1 to 4 V. The inset shows the potential range from -1 to 1.3 V, with oxidation peak at 1.1 V and reduction peak at 0.28 V.

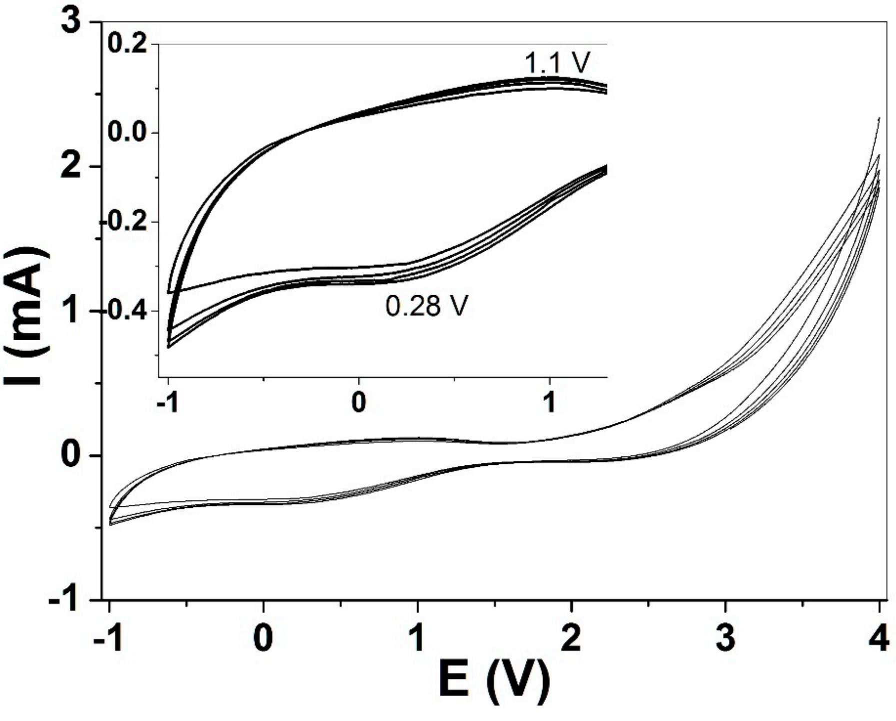

Figure S4. Potentiodynamic polymerization (scan rate 50 mV s^{-1}) of PIL monomer in LiTFSI in propylene carbonate at applied potential range of -1 V to 4 V. The inset shows the potential range from -1 V to 1.3 V, with oxidation peak at 1.1 V and reduction peak at 0.28 V.

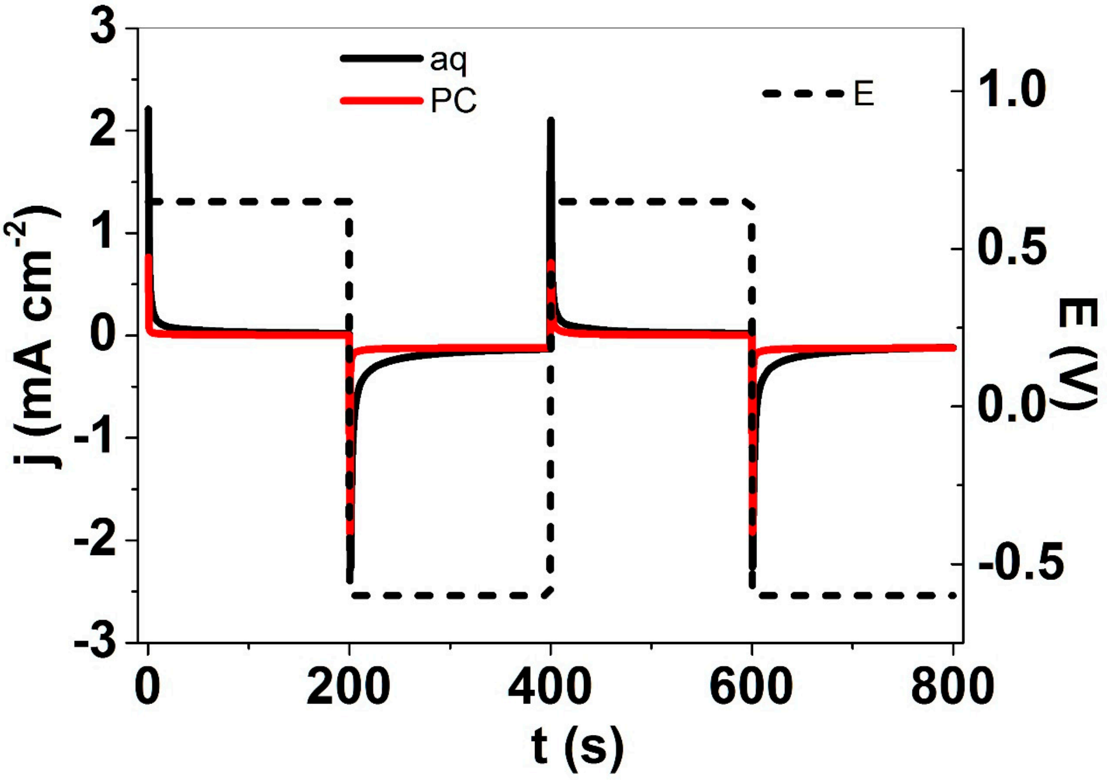

Figure S5. Current density time curve of PIL films in LiTFSI-aq (aq, black line) and LiTFSI-PC (PC, red line), and potential range (dashed line) from 0.65 V to -0.6 V at a frequency of 0.0025 Hz (for the 3rd and 4th cycles).
